# A Survey on the Knowledge, Attitudes, Behaviors and Influencing Factors of Caregivers for Newborns With COVID-19 in Chongqing, China

**DOI:** 10.7759/cureus.53046

**Published:** 2024-01-27

**Authors:** Xiaojun Tao, Yanhan Chen, Ye Xu, Zhengjie Wang, Xuexiu Liu

**Affiliations:** 1 Department of Neonatology, Children’s Hospital of Chongqing Medical University, Chongqing, CHN; 2 College of Nursing, Chongqing Medical University, Chongqing, CHN; 3 Department of Radiology, Children’s Hospital of Chongqing Medical University, Chongqing, CHN; 4 Department of Nuclear Medicine, The First Affiliated Hospital of Chongqing Medical University, Chongqing, CHN

**Keywords:** attitude, influencing factors, behavior, knowledge, caregivers, covid-19

## Abstract

Objective: This study aimed to investigate the healthcare knowledge, attitudes, and behaviors of primary caregivers of newborns with coronavirus disease 2019 (COVID-19) during the pandemic in Chongqing, China, and analyze the influencing factors.

Methods: The study included primary caregivers of COVID-19 newborns hospitalized in our institution from December 2022 to January 2023. A questionnaire survey was initiated to assess the caregivers' health-care knowledge, attitudes, and behaviors for COVID-19 and the influencing factors. The data were analyzed statistically.

Results: A total of 195 caregivers were included, one for each infant with COVID-19. The questionnaire consisted of three dimensions. For the knowledge dimension, the top scoring items were wearing masks in public spaces (4.92 ± 0.087), strengthening hand hygiene (4.83 ± 0.164), and frequent ventilation in living environment (4.62 ± 0.331) in order; for the attitude dimension, the top three scoring items were wearing masks in public spaces (4.85 ± 0.353), strengthening hand washing and disinfection (4.72 ± 0.450), and regular ventilation (4.49 ± 0.501). For the behavior dimension, the top three were confidence in winning the challenge of the pandemic (4.71 ± 0.480), standardized wearing of masks in public spaces/confined spaces (4.68 ± 0.589), and high satisfaction with community epidemic prevention measures (4.67 ± 0.496). Among the influencing factors, fear of COVID-19 was the independent risk indicator for the caregivers' anxiety (OR = 38.085, 95% CI = 14.383-100.664) and fear of COVID-19 (OR = 8.170, 95%CI = 2.156-30.957) and fever (OR = 10.213, 95% CI = 1.972-52.892) were the independent risk indicators for depression.

Conclusion: The study shows a key link between caregiver knowledge, attitudes, behaviors, and neonatal COVID-19 infection, with a gap between knowledge, attitudes, and behaviors. Caregivers, especially those dealing with premature infants, worried about mother-to-child transmission and experienced multiple births, face significant psychological stress during this phase of the pandemic.

## Introduction

Coronavirus disease 2019 (COVID-19) is a highly contagious condition caused by the novel coronavirus. It is characterized by fever, various respiratory symptoms, and sometimes atypical extrapulmonary manifestations [1]. Since December 2019, the COVID-19 virus has rapidly spread globally and continues to mutate, giving rise to multiple strains, including Alpha, Beta, Gamma, Delta, and Omicron, and has become a public health emergency of international concern [2]. The Omicron mutant bears a strong transmission ability, which has been regarded as responsible for the late 2022 pandemic in Chongqing [3].

Newborns, as a special population, are extremely vulnerable to this virus [4]. Once infected, they may be at greater risk for poor outcomes than adult patients [5]. Moreover, the insidious and rapid progression in neonates further complicates the diagnosis and treatment [6]. Since the first case of neonatal COVID-19 was reported in February 2020, new cases are cumulating in China, posing a major challenge for neonatal wards [7]. A couple of recent studies found that parents of newborns in Neonatal Intensive Care Unit (NICU) tended to develop remarkable neuropsychiatric problems including immediate stress, anxiety, depression, post-traumatic stress, and greater adverse parenting outcomes, which have been further aggravated under the COVID-19 pandemic settings [8]. However, the previous literature has focused mainly on the diagnosis and treatment of newborns with COVID-19 [9,10], with little consideration for their caregivers. Caregivers’ knowledge, attitudes, and behaviors for COVID-19 infection have been demonstrated closely relating to prevention and control of the disease as well as recovery and prognosis. For these reasons, we suppose it would be significant to investigate the caregivers’ knowledge, attitudes, and behaviors about COVID-19 and the affecting factors. The findings and results produced would help medical staff provide more efficient help to caregivers involved in troubles.

## Materials and methods

Participants

The study involved caregivers of newborns hospitalized in the institution between December 2022 and January 2023 due to COVID-19. Inclusion criteria for the caregivers were: (1) Being the primary caregiver of a newborn with positive COVID-19 nucleic acid and antigen results; (2) Agreeing to participate in this study with informed consent. Exclusion criteria were: (1) Spending less than half of the hospital days staying with their infants; (2) Unable to finish the study-required tasks due to illness, emotion, or other reasons; (3) Voluntarily withdrawing from the study. The Ethics Committee of Children's Hospital of Chongqing Medical University approved this study with approval no. 2022-559.

Survey tools

A self-designed questionnaire was employed to probe the caregivers’ knowledge, attitude, and behavior in COVID-19-specific health care and the influencing factors. The questionnaire was revised by our research team through more than three circles of discussion and a pilot survey of 10 primary caregivers before being submitted to nursing experts for review to attain the final version. The final version consists of two parts: (1) a questionnaire on basic information, such as gender, age, ethnicity, etc.; (2) a questionnaire to survey the caregivers’ knowledge, attitude, behavior, and the influencing factors. The first questionnaire contains questions about a respondent’s gender, age, nationality, level of education, marital status, religious belief, occupation, place of residence, urban/rural resident, monthly per capita income of the family, relationship with the infant, number of births, full-term or premature, natural or cesarean delivery, among others. The second questionnaire contains 26 questions about the caregiver’s knowledge, attitude, and behavior, including fear, anxiety, depression, knowledge about newborns with COVID-19, transmission ways of COVID-19, when the infant was found having COVID-19, whether the mother was infected with COVID-19, and whether the infant is breastfed, etc.

Expert seminars were called up to review the questionnaire manuscript. Six experts in neonatal medicine with medium-grade professional titles or above, bachelor's degree or above, 10 years of work experience or above, and at least five years of experience in neonatal nursing and management, were invited to form a final draft of 26 items. Then, the content validity of the questionnaire was reevaluated by two neonatal medical experts (chief physicians), two neonatal nursing managers (deputy chief nurses), one medical expert in infectious disease (chief physician), and one nursing manager of infectious disease (deputy chief nurse). The content validity index was 0.94.

To evaluate the status of caregivers’ anxiety and depression, the Self-rating Anxiety Scale (SAS) and Self-rating Depression Scale (SDS) developed by Zung in 1972 were employed [11]. Both scales use a 4-grade scoring method to appraise the frequency of symptoms defined by the items to be appraised. The two scales both contain 20 items, including 10 positive scores and 10 negative scores for each. After self-evaluation, the scores of the 20 items are added to obtain a total score (X), which is then multiplied by 1.25 to get the standard score (Y). A higher Y value represents severe symptoms. According to the Chinese norm, a Y score of ≤ 50, 50-60, 61-70, and > 70 indicates normal, mild, moderate, and severe anxiety for the SAS, respectively; and a Y score of ≤ 53, 53-62, 63-72, and > 73 indicates normal, mild, moderate, and severe depression for the SDS, respectively.

Data collection

One-to-one unified guidance was adopted to explain to the caregivers of newborns about the survey purposes, contents, and respondents, and the qualified respondents were invited to completethe questionnaire. Quality control methods were as follows: (1) anonymous questionnaire; (2) setting an IP address limit to ensure a respondent can only answer once; (3) manual checking to eliminate the questionnaires with regular options. Finally, a total of 195 valid questionnaires were recovered with an effective rate and response rate of 100%.

Statistical analysis

The data that were collected through a mobile-phone-based application software ‘Questionnaire Star’ were imported into Excel, checked, and sorted by two researchers, and then transferred to SPSS software, version 26.0 (IBM Corp., Armonk, NY) and SAS 9.4 (SAS Institute Inc., Cary, NC) for statistical analysis. Measurement data of normal distribution were described by mean ± standard deviation. Counting data were described by frequency, constituent ratio, and percentage. Variables with a P-value<0.05 in univariate analysis were referred for multivariate analysis using a stepwise method for variable screening. The logistic regression model was used to investigate the independent risk factors for the caregivers’ anxiety and depression, while a multiple linear regression model was used to explore the independent risk factors for the caregivers’ knowledge, attitude, and behavior. A P-value<0.05 on both sides was considered significant.

## Results

General information about the caregivers

The general information of the 195 caregivers is minutely shown in Table 1. Of note, 12.8% of the infants present with jaundice as the first symptom; 9.7% were infected by maternity matrons - workers in China who have expertise in infant parenting and are often hired temporarily to support mothers and their newborns. Additionally, 8.2% were infected during hospital examinations such as routine testing for jaundice, vaccination, and follow-up. Mother-to-child transmission of COVID-19 accounted for 9.3% of the pregnant women with pregnancy COVID-19 infection, and 67.5% of the mothers with COVID-19 stopped breastfeeding after delivery. It is worth noting that 76.9% of the caregivers were not aware of drugs such as thymic hormones and human albumin that could promote the body's resistance. Furthermore, 96.9% of the caregivers were only aware of the respiratory route of COVID-19 transmission but not the contact route, such as sharing dishes and chopsticks, inadequately disinfected hands, and unsterilized articles. Finally, 99.4% of the caregivers reported gaining related knowledge mainly from the Internet.

**Table 1:** General information of respondents (n = 195)

Item	Number of persons (n, %)
Gender	
Male	6 (3.1)
Female	189 (96.9)
Age (years)	
≤20	3 (1.5)
20~30	96 (59.2)
30~40	88 (45.1)
≥40	8 (4.1)
Nationality	
Han nationality	191 (98.0)
Others	4 (2.0)
Education	
Junior high school and below	9 (4.6)
High school/vocational school/ technical secondary school	56 (28.7)
College/undergraduate	116 (59.5)
Postgraduate and above	14 (7.2)
Marital status	
Married	192 (98.5)
Unmarried	3 (1.5)
Religious belief	
No	195 (100)
Yes	0
Occupation	
Workers	115 (59.0)
Self-employed	32 (16.4)
Farmer	1 (0.5)
Unemployed	47 (24.1)
Place of residence	
Chongqing	184 (94.4)
Sichuan	10 (5.1)
Others	1 (0.5)
Place of residence	
City	185 (94.9)
Countryside	10 (5.1)
Monthly per capita income of the family	
≤3000	14 (7.2)
3000~5000	33 (16.9)
≥5000	148 (75.9)
Relationship with infant	
Mother	186 (95.4)
Father	5 (2.6)
Grandma	3 (1.5)
Others	1 (0.5)
Birth	
First	126 (64.6)
Second	54 (27.7）
Third	15 (7.7)
Single or multiple	
Singleton	175 (89.8)
Twins	18 (9.2)
Triplets	2 (1.0)
Full term or premature	
Term infant	141 (72.3)
Premature infant	54 (27.7)
Natural delivery or C-section	
Natural delivery	94 (48.2)
Cesarean section	101 (51.8)

Anxiety and depression of the caregivers

According to the SAS scoring and SDS scoring, 44 (22.6%) of the caregivers were affected by anxiety of different degrees, and 25 (12.8%) were affected by depression (Table 2).

**Table 2:** Anxiety and depression of caregivers (n = 195)

Item	Number of cases (n, %)	Score (x± s)
Anxiety		
No	151 (77.5)	31.49±5.67
Mild	23 (11.8)	55.15±3.02
Moderate	19 (9.7)	64.61±2.24
Severe	2 (1.0)	73.75±0.00
Depression		
No	170 (87.2)	31.47±4.87
Mild	24 (12.3)	56.63±3.58
Moderate	1 (0.5)	65.00±0.00
Severe	0	

Risk factor analysis of the caregivers' SAS results

To investigate the significant factors correlated with the caregivers’ SAS results, univariate analyses were performed for the factors including gender, age, education level, occupation, urban/rural residents, income, number of births, single/multiple births, full-term or premature, natural or cesarean delivery, afraid of COVID-19, and mother with or without COVID-19. Among these factors, single/multiple births (P=0.005), full-term or premature (P=0.002), and afraid of COVID-19 (P<0.001) showed a significant correlation with the caregiver's anxiety. A binary logistic regression analysis was used to probe the independent risk factors using a stepwise method, with anxiety as the dependent variable and the three factors as independent variables. The variables were assigned a value of yes = 1, no = 0. The results showed that only the fear of COVID-19 (OR = 38.051, 95% CI = 14.383-100.664) had a significant influence on the occurrence of caregivers' anxiety (Table 3). 

**Table 3:** Single factor and multiple factor analysis of caregivers' SAS (n = 195) SAS: Self-rating Anxiety Scale

Item	Total	SAS		χ2	P	Multivariate logistic regression
		No (n=151)	Yes (n=44)			β Standard error Waldχ2 P OR (95%CI)
Gender						
Male	6 (3.08)	6 (100.00)	0 (0.00)	/	0.599	
Female	189 (96.92)	151 (79.89)	38 (20.11)			
Place of residence						
City	185 (94.87)	150 (81.08)	35 (18.92)	/	0.413	
Countryside	10 (5.13)	7 (70.00)	3 (30.00)			
Income						
≤5000	47 (24.10)	40 (85.11)	7 (14.89)	0.833	0.361	
>5000	148 (75.90)	117 (79.05)	31 (20.95)			
Birth						
First	126 (64.62)	101 (80.16)	25 (19.84)	0.028	0.866	
Second/Third	69 (35.38)	56 (81.16)	13 (18.84)			
Single or multiple						
Singleton	175 (89.74)	146 (83.43)	29 (16.57)	/	0.005	1.0 (Singleton)
Twins/Triplets	20 (10.26)	11 (55.00)	9 (45.00)			1.314 0.695 3.571 0.059 3.722 (0.952,14.545)
Full term or premature						
Term infant	141 (72.3）	135 (95.74)	6 (4.26)	/	0.002	1.0 (Term infant)
Premature infant	54 (27.7）	16 (29.63)	38 (70.37)			1.675 1.138 2.166 0.141 5.337 (0.574,49.652)
Natural delivery or cesarean section						
Natural delivery	94 (48.21)	81 (86.17)	13 (13.83)	3.702	0.054	
Cesarean section	101 (51.79)	76 (75.25)	25 (24.75)			
Fear of COVID-19						
No	154 (78.97)	145 (94.16)	9 (5.84)			1.0 (No)
Yes	41 (21.03)	12 (29.27)	29 (70.73)	86.892	<0.001	3.639 0.496 53.746 <0.001 38.051 (14.383,100.664)
COVID-19 Symptom - Fever						
No	114 (58.46)	94 (82.46)	20 (17.54)	0.661	0.416	
Yes	81 (41.54)	63 (77.78)	18 (22.22)			
COVID-19 Symptom - Pharyngeal Pain						
No	186 (95.38)	151 (81.18)	35 (18.82)	/	0.381	
Yes	9 (4.62)	6 (66.67)	3 (33.33)			
Developing COVID-19 in utero/after birth						
Intrafetal transmission	18 (9.23)	13 (72.22)	5 (27.78)	/	0.354	
Postnatal	177 (90.77)	144 (81.36)	33 (18.64)			
Mother infected with COVID-19						
No	173 (88.72)	140 (80.92)	33 (19.08)	/	0.775	
Yes	22 (11.28)	17 (77.27)	5 (22.73)			

Risk factor analysis of the caregivers' SDS results

To investigate the significant factors correlated with the caregivers’ SDS results, univariate analyses were also performed for the factors including the first 11 in SDS and five additional factors (with/without fever, with/without cough, with/without fatigue, with/without sore throat, and developing COVID-19 in utero/after birth). Among these factors, urban/rural residents (P = 0.032), full-term or premature birth (P = 0.009), natural delivery or C-section (P = 0.007), caregivers with/without fever (P < 0.001), caregivers with/without pharyngeal pain (P = 0.024), and developing COVID-19 in utero/after birth (P = 0.037) exhibited significant correlation with the respondent’s depression. A binary logistic regression analysis was conducted using a stepwise method, with depression as the dependent variable and the significance factors as independent variables. The variables were assigned a value in advance: yes = 1, no = 0. Resultantly, two factors, afraid/not afraid of COVID-19 (OR = 8.170, 95% CI = 2.156-30.957) and caregiver with/without fever (OR = 10.213, 95% CI = 1.972-52.892) showed significant influence on the development of caregivers' depression (Table 4).

**Table 4:** Single factor and multiple factor analysis of caregivers' SDS (n = 195) SDS: Self-rating Depression Scale

Item	Total	SDS		χ2	P	Multivariate logistic regression
		No (n=170)	Yes (n=25)			β Standard error Waldχ2 P OR (95%CI)
Gender						
Male	6 (3.08)	6 (100.00)	0 (0.00)	/	>0.999	
Female	189 (96.92)	174 (92.06)	15 (7.94)			
Place of residence						
Countryside	10 (5.13)	7 (70.00)	3 (30.00)	/	0.032	1.0 (Countryside)
City	185 (94.87)	173 (93.51)	12 (6.49)			0.979 1.143 0.734 0.392 2.662 (0.283,25.031)
Income						
≤5000	47 (24.10)	43 (91.49)	4 (8.51)	/	0.760	
>5000	148 (75.90)	137 (92.57)	11 (7.43)			
Birth						
First	126 (64.62)	117 (92.86)	9 (7.14)	0.151	0.697	
Second/Third	69 (35.38)	63 (91.30)	6 (8.70)			
Single or multiple						
Singleton	175 (89.74)	161 (92.00)	14 (8.00)	/	>0.999	
Twins/Triplets	20 (10.26)	19 (95.00)	1 (5.00)			
Full term or premature						
Term infant	141 (72.3）	135 (95.74)	6 (4.26)	/	0.009	1.0 (Term infant)
Premature infant	54 (27.7）	35 (64.81)	19 (35.19)			1.675 1.138 2.166 0.141 5.337 (0.574,49.652)
Natural delivery or C-section						
Natural delivery	94 (48.21)	92 (97.87)	2 (2.13)	7.282	0.007	1.0 (Natural delivery)
Cesarean section	101 (51.79)	88 (87.13)	13 (12.87)			1.612 0.845 3.641 0.056 5.014 (0.957,26.266)
Fear of COVID-19						
No	41 (21.03)	32 (78.05)	9 (21.95)	/	0.001	1.0 (No)
Yes	154 (78.97)	148 (96.10)	6 (3.90)			2.100 0.680 9.551 0.002 8.170 (2.156,30.957)
COVID-19 Symptom - Fever						
No	114 (58.46)	112 (98.25)	2 (1.75)	12.797	<0.001	1.0 (No)
Yes	81 (41.54)	68 (83.95)	13 (16.05)			2.324 0.839 7.669 0.006 10.213 (1.972,52.892)
COVID-19 Symptom - Pharyngeal Pain						
No	186 (95.38)	174 (93.55)	12 (6.45)	/	0.024	1.0 (No)
Yes	9 (4.62)	6 (66.67)	3 (33.33)			1.675 1.138 2.166 0.141 5.337 (0.574,49.652)
Developing COVID-19 in utero/after birth						
Postnatal	177 (90.77)	166 (93.79)	11 (6.21)	/	0.037	1.0 (Postnatal)
Intrafetal transmission	18 (9.23)	14 (77.78)	4 (22.22)			0.345 0.955 0.131 0.718 1.412 (0.217,9.178)
Mother infected with COVID-19						
No	173 (88.72)	158 (91.33)	15 (8.67)	/	0.226	
Yes	22 (11.28)	22 (100.00)	0 (0.00)			

Scores of the knowledge, attitudes, and behaviors of the caregivers 

The scores of the 31 items in the three dimensions, knowledge, attitudes, and behaviors were summarized. Wearing masks in public places and confidence in overcoming the struggles of the pandemic got the highest score separately, as shown in Table 5.

**Table 5:** Score of knowledge, attitudes, and behaviors of caregivers (n = 195)

Item	Score
Knowledge	
Wear masks in public places	4.92±0.087
Strengthen hand hygiene	4.83±0.164
Frequent ventilation of living environment	4.62±0.331
Frequent disinfection	4.35±0.275
Wear gloves	4.27±0.267
Use drugs to prevent COVID-19	4.17±0.375
Exercise to enhance immunity	4.15±0.281
Monitoring body temperature	4.13±0.334
Correct treatment of domestic garbage and secretions: seal after alcohol spraying	3.91±0.431
Attitudes	
Wear masks in public places	4.85±0.353
Strengthen hand washing and disinfection	4.72±0.450
Regular ventilation	4.49±0.501
Avoid activities in crowded places	4.34±0.476
Keep a safe social distance (1-2m)	4.32±0.468
Avoid contact with respiratory tract patients such as influenza symptoms or pneumonia	4.31±0.465
Cover mouth and nose when coughing or sneezing	4.26±0.441
Check the temperature of the whole people	4.26±0.441
Preventive medication	4.02±0.621
Behaviors	
Confidence in overcoming the struggles of the pandemic	4.71±0.480
Wear masks in public places/confined spaces	4.68±0.589
High satisfaction with community epidemic prevention measures	4.67±0.496
Keep hands clean and wash hands properly	4.65±0.557
Cooperate with epidemic prevention management (temperature measurement and code scanning, etc.)	4.48±0.577
Return from outside, wash hands before and after meals	4.44±0.604
Frequently open windows for ventilation in residence/office	4.42±0.725
Cover mouth and nose with coughing or sneezing tissue	4.41±0.571
Keep a safe distance from others	4.36±0.674
Reduce the number of parties, dinners and business trips	4.35±0.681
Pay close attention to the physical condition and check the temperature	4.28±0.687
Disinfect yourself and the room	4.26±0.728
Dissuade friends from gathering and other high-risk behaviors	4.16±0.752

Linear regression analysis of the knowledge, attitudes, and behaviors of the caregivers

Table 6 presents the results of statistical analyses conducted for the three dimensions of knowledge, attitudes, and behaviors. Univariate analysis revealed that various factors such as age, education level, occupation, place of residence, income, anxiety, depression when the baby was infected with COVID-19, where the baby was infected with COVID-19, where the baby lived when infected with COVID-19, whether the caregiver knows the transmission routes of COVID-19, whether the caregiver knew the drugs that increase immune resistance, whether the caregiver often disinfected the home environment, whether the caregiver wore masks and washed or disinfected hands before contact with the baby, were significantly correlated to the caregivers’ knowledge, attitudes, behaviors. The study found that the knowledge and attitudes scores of the caregivers of infants infected in hospitals or at maternity centers were significantly higher than those of infants infected at home (40.76 ± 3.17 vs. 38.95 ± 3.03, P<0.001). A multiple linear regression analysis was performed to investigate independent risk factors, with caregivers' knowledge, attitudes, and behavior scores as the dependent variables and significant variables from univariate analyses as independent variables. The results are presented in Table 7. Resultantly, the factors, including income, anxiety, occupation, place of residence, whether the caregiver knows the transmission route of COVID-19, whether the caregiver knows the drugs that increase immune resistance, whether the caregiver disinfected the home environment, whether the caregiver wore masks and washed or disinfected hands before contact with the baby, were the independent risk ones of caregivers' knowledge, attitudes, and behaviors.

**Table 6:** Single factor analysis of influencing factors on caregiver's knowledge, attitudes, and behaviors scores (n = 195)

Item	n (%)	Knowledge	P	Attitude	P	Behavior	P
Age							
≤30	99 (50.77)	39.65±3.27	0.507	34.91±2.89	0.039	57.10±6.80	0.048
>30	96 (49.23)	39.34±3.08		35.76±2.83		58.83±5.27	
Education							
High school and below	65 (33.33)	37.97±2.70	<0.001	34.03±2.52	<0.001	55.66±7.33	0.001
Bachelor's degree or above	130 (66.67)	40.26±3.13		35.98±2.84		59.10±5.10	
Occupation							
Unemployed	47 (24.10)	37.79±2.32	<0.001	33.77±2.16	<0.001	55.77±7.21	0.015
Employed	148 (75.90)	40.04±3.22		35.82±2.91		58.65±5.60	
Place of residence							
City	185 (94.87)	39.69±3.07	<0.001	35.42±2.84	0.067	58.10±6.09	0.146
Countryside	10 (5.13)	36.00±3.27		33.70±3.30		55.20±6.78	
Income							
≤5000	47 (24.10)	37.45±2.83	<0.001	33.94±2.61	<0.001	55.45±7.05	0.005
>5000	148 (75.90)	40.15±3.00		35.77±2.83		58.75±5.61	
Anxious							
No	157 (80.51)	39.52±3.01	0.887	35.31±2.81	0.826	57.50±6.13	0.034
Yes	38 (19.49)	39.42±3.83		35.42±3.22		59.84±5.88	
Depressed							
No	180 (92.31)	39.67±3.10	0.010	35.39±2.86	0.310	57.86±6.05	0.440
Yes	15 (7.69)	37.47±3.50		34.60±3.11		59.13±7.28	
Developing COVID-19 in utero/after birth							
Postnatal	18 (9.23)	39.28±3.83	0.759	35.17±3.42	0.804	60.78±5.66	0.040
Intrafetal transmission	177 (90.77)	39.52±3.11		35.34±2.83		57.67±6.13	
The place where the baby gets the COVID-19							
At home	136 (69.74)	38.95±3.03	<0.001	34.99±2.65	0.013	57.50±6.31	0.117
In hospital	59 (30.26)	40.76±3.17		36.10±3.26		59.00±5.63	
Baby's place of residence							
At home	170 (87.18)	39.12±3.01	<0.001	35.00±2.71	<0.001	57.34±6.11	<0.001
Other	25 (12.82)	40.08±3.13		37.56±3.07		62.12±4.61	
Know the transmission ways of COVID-19							
Yes	69 (35.38)	41.13±3.56	<0.001	36.78±3.24	<0.001	60.68±5.67	<0.001
No	126 (64.62)	38.60±2.54		34.53±2.32		56.46±5.89	
Know (inject) drugs that increase body resistance							
Yes	45 (23.08)	41.96±2.95	<0.001	37.38±2.88	<0.001	62.82±3.64	<0.001
No	150 (76.92)	38.76±2.86		34.71±2.59		56.49±5.99	
Frequently disinfect the home environment							
Yes	151 (77.44)	40.07±2.91	<0.001	35.65±2.91	0.004	58.74±5.80	0.001
No	44 (22.56)	37.55±3.32		34.23±2.51		55.27±6.58	
Wear a mask when touching the baby							
Yes	175 (89.74)	39.75±3.13	0.001	35.51±2.92	0.001	58.35±5.84	0.008
No	20 (10.26)	37.25±2.75		33.70±1.89		54.50±7.63	
Wash hands or disinfect hands before touching the baby							
Yes	180 (92.31)	39.68±3.10	0.004	35.51±2.92	<0.001	58.32±5.98	0.003
No	15 (7.69)	37.27±3.31		33.13±0.83		53.53±6.44	

**Table 7:** Multivariate linear regression analysis of the knowledge, attitudes, and behaviors score of caregivers (n = 195)

Dependent variable	Independent variable	β	Standard error	Standardized regression coefficient	t	P
Knowledge	Constant	34.419	0.865	—	39.787	<0.001
	Income	2.421	0.840	0.169	2.884	0.004
	Place of residence	0.965	0.464	0.130	2.079	0.039
	COVID-19 Symptoms - Pharyngeal Pain	-2.627	0.904	-0.174	-2.907	0.004
	Know the transmission way of COVID-19	1.737	0.417	0.262	4.161	<0.001
	Know (inject) drugs to increase body resistance	1.838	0.466	0.244	3.940	<0.001
	Disinfection of the home environment	1.461	0.455	0.193	3.212	0.002
Attitudes	Constant	32.189	0.604	—	53.318	<0.001
	Occupation	1.108	0.429	0.165	2.584	0.011
	Know the transmission route of COVID-19	1.264	0.396	0.210	3.194	0.002
	Know (inject) drugs to increase body resistance	1.839	0.448	0.269	4.108	<0.001
	Contact baby wearing a mask	1.361	0.585	0.144	2.327	0.021
	COVID-19 Symptoms - Cough	0.884	0.365	0.151	2.425	0.016
	Baby symptoms - feeding intolerance	-1.290	0.543	-0.145	-2.373	0.019
Behaviors	Constant	52.115	1.006	—	51.804	<0.001
	Know the transmission route of COVID-19	2.279	0.840	0.178	2.714	0.007
	Know (inject) drugs to increase body resistance	4.812	0.963	0.331	4.997	<0.001
	Frequently disinfect the home environment	2.668	0.912	0.182	2.925	0.004
	Anxious	1.980	0.945	0.128	2.095	0.038
	Baby symptoms - fever	2.257	0.789	0.176	2.862	0.005

## Discussion

Influence of caregivers’ socio-demographic features on their knowledge, attitudes, and behaviors

This study discovered that the knowledge, attitudes, and behaviors of the caregivers were significantly affected by various socio-demographic factors, including age, education level, occupation, place of residence, income, anxiety and depression status, COVID-19 symptoms, and where their infant stays after birth. Caregivers who were younger had higher education levels, held stable jobs, lived in urban areas, did not display COVID-19 symptoms, and had their infants stay in hospitals or commercial newborn-care centers after birth tended to exhibit a higher level of knowledge, attitudes, and behaviors during healthcare. These results may be attributed to the advantages that younger individuals have in utilizing learning resources, absorbing new knowledge, possessing modern information tools, and having effective social circles [12]. Additionally, the study also found that for families employing nannies, COVID-19 was transmitted mainly through the nannies (9.7%). This could be partly due to the nannies' insufficient infection-prevention knowledge and skills.

Performance of the caregiver in knowledge, attitudes, and behaviors

According to the knowledge, attitudes, and behaviors theoretical model, changing human behavior involves three sequential steps: acquiring knowledge, generating attitudes, and then forming behaviors. Realization of the ultimate goal ‘behaviors’ needs a sufficient preparation of knowledge and positive attitudes, with belief as the pivot [13]. As individuals’ knowledge accumulates, their attitudes and behaviors change accordingly [14]. In this study, the knowledge dimension scored the highest (4.92 ± 0.087), whereas the behavior dimension was the lowest (4.71 ± 0.480). The findings underscore the significance of sufficient knowledge and attitude preparation for extraordinary behaviors. Therefore, we believe that providing sufficient and effective medical education to caregivers is essential for obtaining satisfactory baby care skills during epidemics.

Effects of pre-hospitalization health education on caregivers’ knowledge, attitudes, and behaviors

In terms of knowledge preparation, this study showed that deficits existed although the respondents perform generally well. For instance, 9.3% of pregnant women contracted COVID-19 during pregnancy due to a lack of prophylactic vaccination before conceiving, leading to mother-to-child transmission. To avoid this, it is strongly recommended that pregnant mothers receive prophylactic vaccination to safeguard both themselves and their newborns from COVID-19 infection [15]. This study also found that the majority of pregnant women (58.9%), after contracting COVID-19, stopped breastfeeding, although breastfeeding is encouraged in COVID-19 cases [16]. It is worth noting that drugs such as thymus hormone and human albumin are known to significantly improve human immunity [17]. However, 76.9% of the caregivers were unaware of this. Furthermore, 96.9% of the caregivers only knew the COVID-19 virus was airborne but did not know that it could also be transmitted through contact, such as by sharing dishes and chopsticks or by using public living articles [18]. Regarding behavior, the respondents generally perform poorly, e.g., failing to cover their mouth and nose while coughing or sneezing (4.41 ± 0.57), which is consistent with the findings of other authors [19]. These findings suggest that the caregivers still lack knowledge about infection prevention and control, thus manifesting as neglect of home disinfection (4.35 ± 0.275), home ventilation (4.62 ± 0.331), and closed treatment of secretions (3.91 ± 0.431), etc. According to traditional Chinese beliefs, ventilation and cold should be prevented for the mothers and children during confinement during childbirth. Therefore, we recommend that public media, especially online media, reinforce the publicity of COVID-19 prevention knowledge and skills [20], significantly improving people's prevention ability [21].

Effects of hospital health education on caregivers’ knowledge, attitudes, and behaviors

This study showed that the knowledge and attitudes of the caregivers of the infants infected during hospitalization and staying at maternity centers scored significantly higher than those of the infants infected at home (40.76 ± 3.17 vs. 38.95 ± 3.03, P<0.001), consistent with Maroiu et al. 's study [22]. This result highlights the significance of medical education programs on the caregivers’ knowledge, attitudes, and behaviors. During the COVID-19 epidemic, hospital-provided health education programs have shown to be helpful in changing people's knowledge and attitudes, thus improving caregivers’ care ability [23]. In addition, neonatal COVID-19 symptoms are often subtle, and caregivers may mistake them for normal and overlook the need for early treatment. Therefore, medical professionals have a responsibility to spread COVID-19-related knowledge to caregivers [24].

Factors affecting caregivers’ anxiety and depression

Anxiety and depression are common mental problems that can lead to abnormal functions in the hypothalamic-pituitary-adrenal tissue and subsequent impairment of the glucocorticoid-receptor-rich hippocampus, resulting in abnormal behaviors [25]. This study showed that 95.3% of the caregivers were mothers with an incidence of 22.6% (44/195) and 12.8% (25/195) separately for anxiety and depression. This may relate to the physical and psychological characteristics of women and the excessive burden of care they take on. Pregnant women, in particular, may be susceptible to anxiety, depression, and other stresses during postpartum recovery [26]. Therefore, medical staff should be attentive to caregivers' needs for information, communicate with them, promote their positive knowledge, attitudes, and behaviors, if necessary, provide them with counseling to relieve anxiety and depression [27,28]. In addition, the study showed significant differences between the caregivers with depression and those without depression in the factors as follows: place of residence, single/multiple birth, full-term/premature birth, vaginal/cesarean delivery, afraid/not afraid of COVID-19, and whether it is transmitted in utero. Among them, fear/not being afraid of COVID-19 and COVID-19 symptoms presented significant impacts on the caregivers' depression. The study also showed significant differences between the caregivers with anxiety and those without anxiety in the factors as follows: single/multiple birth, full-term/premature birth, and afraid/not afraid of COVID-19, among which the item afraid/not afraid of COVID-19 has a significant effect on caregivers' anxiety. In fact, the association of multiple births, premature infants, cesarean delivery, and mother-to-child transmission, with anxiety and depression have been well documented in previous studies [29]. In addition to these findings, our current study further revealed that 67.5% of mothers with COVID-19 stopped breastfeeding after delivery, and mother-infant separation aggravated their anxiety and depression. Therefore, the importance of breastfeeding and kangaroo care should be emphasized to alleviate anxiety and depression [30].

Limitations

This study has some limitations that need to be considered. Firstly, the research was carried out in a single-center setting, specifically within the Chongqing region, which may not represent the broader population’s experiences and limit the generalizability of the findings. Secondly, due to the limited number of caregivers of infants with COVID-19, only a small sample size was studied. Thirdly, the WHO chief declared the end to COVID-19 as a global health emergency on 5th May 2023; the pandemic lasting only several months this study did not conduct continuous follow-ups on the caregivers. Finally, the knowledge, attitudes, behaviors, and anxiety have changed over the three-year pandemic, especially since the study was conducted during the Omicron variant. Therefore, additional research in other settings is needed in order to determine whether our findings are generalizable.

## Conclusions

This study reveals a crucial link between caregivers' knowledge, attitudes, behaviors, and neonatal COVID-19 infection. Despite high levels of knowledge and positive attitudes, caregivers often exhibit suboptimal practical behaviors. Furthermore, some caregivers experienced high levels of anxiety, depression, and psychological pressure, particularly those who were afraid of COVID-19, had premature infants, worried about mother-to-child transmission, and experienced multiple births. Medical staff should be aware of these findings and provide effective and timely support to caregivers.
